# Invasive Fungal Sinusitis Minimally Evident by Physical Examination

**DOI:** 10.5811/cpcem.2018.4.37872

**Published:** 2018-05-18

**Authors:** Manish Amin, Vikram S. Shankar, Laura C. Castro, Phillip Aguìñiga-Navarrete

**Affiliations:** Kern Medical, Department of Emergency Medicine, Bakersfield, California

## CASE PRESENTATION

**Image 1:**
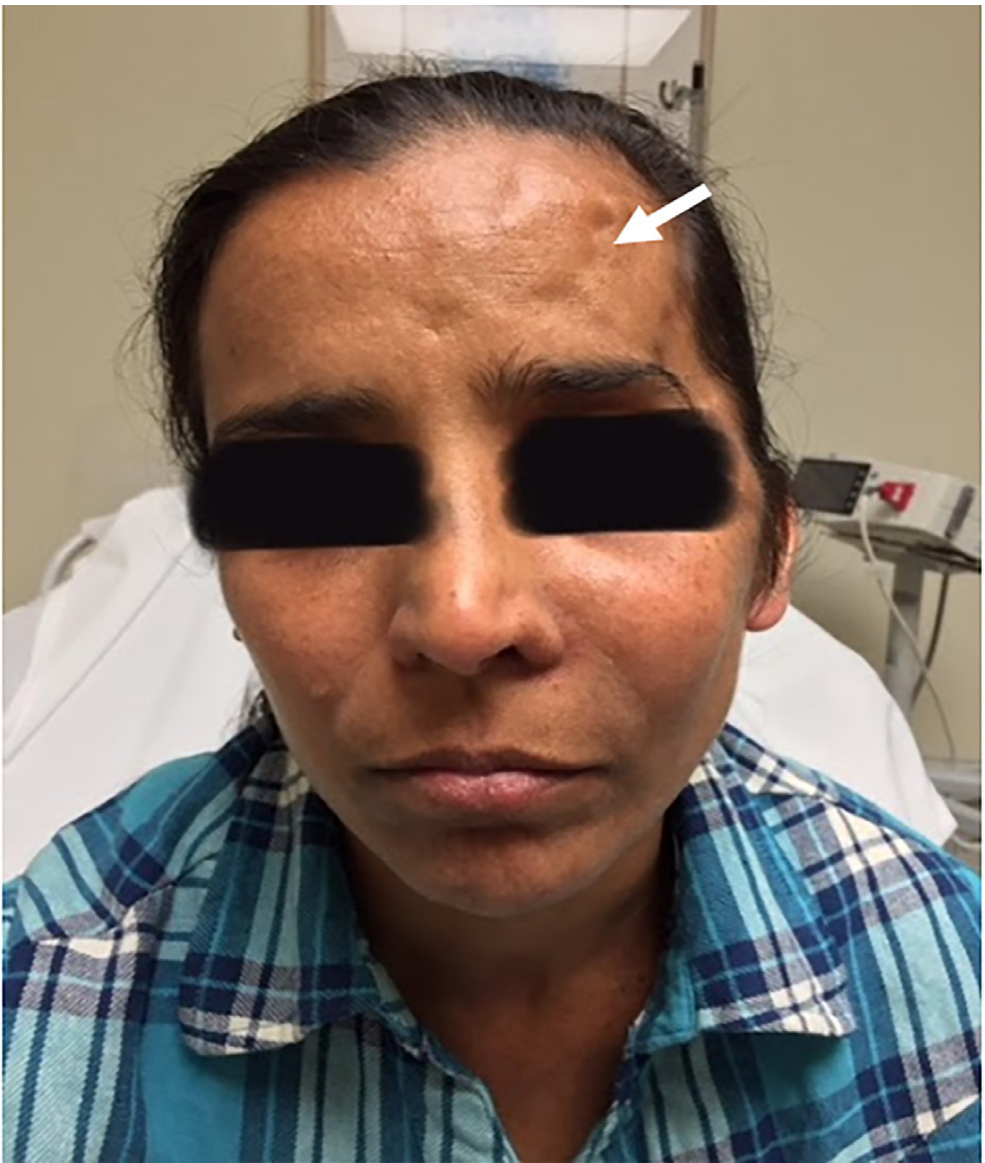
Photograph of patient with minimally evident presentation of invasive fungal infection and old fibrotic scar (white arrow).

A 35-year-old immunocompetent female with a history of intracranial fungal abscess with surgical resection 11 years prior presented with headache for four months. Her headache was located along frontal sinuses. Vital signs were normal. Head examination was significant for minimal left maxillary swelling with mild tenderness to palpation (Image 1). A fibrotic scar located on the right forehead was present from previous craniectomy. Nasal turbinates were normal appearing. Neurologic examination was normal.

**Image 2:**
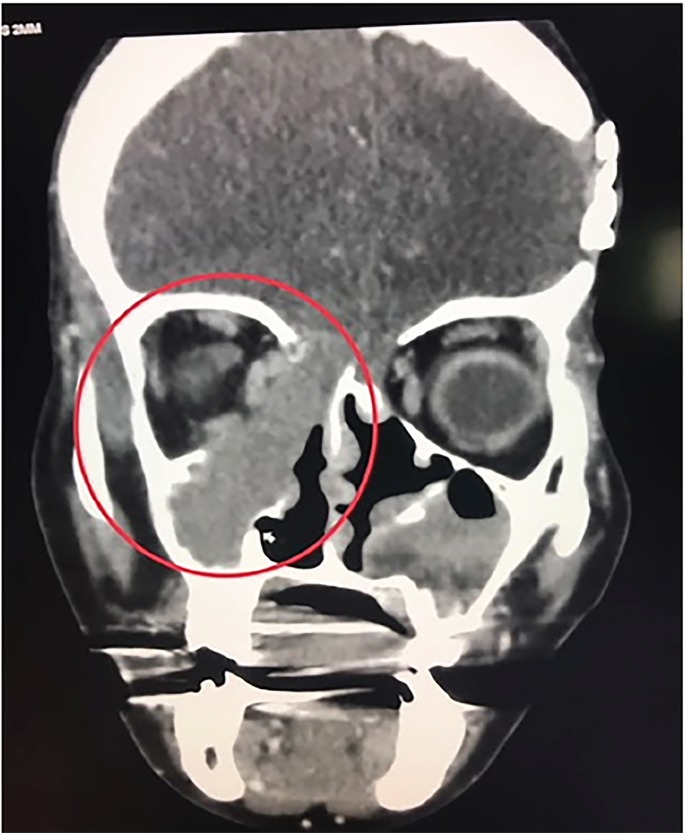
Right-sided mass with extension into ethmoid and maxillary sinuses (red circle).

Complete blood count and electrolytes were within normal limits. Computed tomography of the face showed ethmoid and maxillary sinus bone destructions with extension into the right frontal lobe and surrounding facial structures, consistent with severe fungal disease (Image 2). Inpatient nasal endoscopy with biopsy showed fungal elements consistent with Aspergillus species.

## DISCUSSION

Aspergillus species, Fusarium species, the Mucorales, and dematiaceous (brown-black) molds are among the most common causative agents of invasive fungal sinusitis.^1^,^2^ The chronic course is typically greater than 12 weeks and takes an indolent form that may present with little or no systemic signs or symptoms.^3^,^4^ Therefore, the emergency physician must maintain a high index of suspicion for such pathology. In the case of our patient, the extensive and severe nature of her pathology was not appreciated by physical examination. Physical exam should include careful inspection of the nares and oral cavity for areas of necrosis.^5^ Other physical exam findings may include tenderness to palpation of the maxillary sinuses. Neurologic examination may reveal decreased sensation in malar areas and visual changes due to optic nerve and/or orbit involvement.

In general, invasive rhinosinusitis is difficult to cure and survival rates are poor. Long-term sinonasal complications such as mycotic aneurysms, cavernous sinus thrombosis, and cerebral infarcts or hemorrhage may develop.^6^ Because of the poor prognosis, early diagnosis and aggressive treatment is necessary. A high index of suspicion for invasive fungal infection should be maintained in patients complaining of sinus symptoms including facial pain and headache, especially in the setting of immunocompromised status.

**CPC-EM Capsule**

What do we already know about this clinical entity?

Aspergillus, Fusarium, and Mucorales are the most common causative agents of invasive fungal sinusitis. Computed tomography specificity and sensitivity is not optimal for diagnosis.

What is the major impact of the image(s)?

The images demonstrate how a chronic course may take an indolent form and present with little or no physical signs, and that aggressive treatment may be necessary given potentially benign findings.

How might this improve emergency medicine practice?

These images raise awareness of the need to maintain a high index of suspicion of a potentially acute disease that may be difficult to diagnose clinically, but where early intervention may be life-saving.

Documented patient informed consent and/or Institutional Review Board approval has been obtained and filed for publication of this case report.
